# Electromagnetic, Air and Fat Frying of Plant Protein-Based Batter-Coated Foods

**DOI:** 10.3390/foods12213953

**Published:** 2023-10-29

**Authors:** Md. Hafizur Rahman Bhuiyan, Michael O. Ngadi

**Affiliations:** Department of Bioresource Engineering, McGill University, Sainte-Anne-de-Bellevue, QC H9X 3V9, Canada; md.bhuiyan@mail.mcgill.ca

**Keywords:** meat analog, mass exchange, hot air, infrared, microwave, microstructure

## Abstract

There is growing consumer and food industry interest in plant protein-based foods. However, quality evolution of plant protein-based meat analog (MA) is still a rarely studied subject. In this study, wheat and rice flour-based batter systems were used to coat plant protein-based MA, and were partially fried (at 180 °C, 1 min) in canola oil, subsequently frozen (at −18 °C) and stored for 7 days. Microwave heating (MH), infrared heating (IH), air frying (AF) and deep-fat frying (DFF) processes were employed on parfried frozen MA products, and their quality evolution was investigated. Results revealed that the fat content of MH-, IH- and AF-treated products was significantly (*p* < 0.05) lower than DFF-treated counterparts. Batter coatings reduced fat uptake in DFF of MA-based products. Both the batter formulations and cooking methods impacted the process parameters and quality attributes (cooking loss, moisture, texture, color) of MA-based coated food products. Moreover, the post-cooking stability of moisture and textural attributes of batter-coated MA-based products was impacted by both the batter formulations and cooking methods. Glass transition temperature (Tg) of MA-based products’ crust ranged from −20.0 °C to −23.1 °C, as determined with differential scanning calorimetry. ATR-FTIR spectroscopy and scanning electron microscopy analysis revealed that surface structural–chemical evolution of MA-based products was impacted by both the coating formulations and cooking methods. Overall, AF has been found as a suitable substitute for DFF in terms of studied quality attributes of meat analog-based coated products.

## 1. Introduction

Due to various reasons (health, ethics, religion, animal welfare, natural resources, greenhouse gas emission, market price, etc.) the demand for animal meat alternatives is continually increasing, and in this context, plant protein-based meat analog (MA) is getting special attention [1,2,3,4,5]. Like other typical food items, MA-based food products require cooking/heating before consumption. Frying is a very fast and popular food preparation method in which foods are cooked via heating within edible oil [6,7]. However, the presence of a high amount of fat in fried products is a major drawback from a health point of view. Additionally, the frying process requires a huge amount of oil (as a cooking medium) which is not appreciable in the food processing industry from a financial point of view. In these circumstances, food surface modification in terms of batter coatings could be an effective strategy, as frying (causing moisture loss and fat uptake) is majorly a surface phenomenon, and batter coating creates a uniform layer over food surfaces that could influence heat and mass transfer processes [8,9]. However, parfrying (PF) is a unit operation where foods are partially cooked via heating in edible oil at a high temperature to inactivate enzymes and microbes [10]. At commercial and home level, parfried products are generally stored frozen, and they require finish-cooking before consumption [7,10,11]. Parfried frozen products such as chicken nuggets, fish strips, etc., are finish-cooked (heated up) mainly by deep-fat frying (DFF). The two-time fat frying (PF, DFF) process results in the presence of an excessively high amount of fat in finish-cooked products [7]. In these scenarios, the use of an alternative heating technique to DFF (as the finish-cooking method) would be a rational strategy to cook plant protein-based parfried frozen products.

The electromagnetic spectrum between the frequencies of 300 MHz and 300 GHz represent microwaves, MW (Singh & Heldman, 2009). MW heating possesses many advantages such as rapid temperature generation, shorter treatment time and improving product uniformity [12,13]. Infrared (IR) is part of the electromagnetic spectrum in the range of 0.5–100 μm, and is absorbed by food compounds which results in temperature rise [14]. Air frying (AF) is an emerging cooking technique where hot air is used as a heat transfer medium instead of edible oil [15]. Recent studies reported that microwave and air frying results in less oil deterioration and reduces acrylamide (human carcinogen) formation in fried products [15,16,17]. It is well understood that these (MW, IR, AF) emerging heating techniques possess many advantages over conventional deep-fat frying (DFF). However, scientific study relating to the impacts of these emerging techniques on the quality evolution of plan protein-based batter-coated parfried frozen products, is not available yet. It could be hypothesized that the evolution of plant protein-based batter-coated parfried frozen product quality would be greatly impacted by the finish-cooking methods. 

Objectives of this study were (i) to assess the impacts of electromagnetic (MW, IR) heating, air frying and deep-fat frying on the quality evolution of plant protein-based batter-coated parfried frozen products (ii) to study the effects of coating formulation and cooking methods on the post-cooking stability of major quality attributes of plant protein-based coated products. In this study, plant protein-based laboratory-formulated meat analog (MA) was used as a model food system.

## 2. Materials and Methods

Soy protein isolate was purchased from MP Biomedicals (29525 Fountain Pkwy, Solon, OH, USA). Wheat gluten was supplied by Sigma-Aldrich Co. (Oakville, ON, Canada). Methylcellulose (M352-500, 4000 centipoises) and sodium pyrophosphate (Na_4_P_2_O_7_, S390, F.W. 446.06) crystals were received from Fisher Scientific (Fair Lawn, NJ, USA). Sodium bicarbonate (NaHCO_3_) was supplied by Church & Dwight Canada Corp. (Toronto, ON, Canada). Rice flour (Suraj^®^, composed of 6.67% protein, 83.32% carbohydrate, 1.33% fat and 8.68% moisture) was procured from a grocery store in Montreal, Canada. Commercial wheat flour (Five Roses^®^, composed of 13.33% protein, 73.33% carbohydrate, 3.33% fiber, 1.33% fat and 8.68% moisture), NaCl (Sifto, Compass Minerals Canada Corp., Toronto, ON, Canada) and canola oil (Sans nom^®^, Loblaws Inc., Toronto, ON, Canada) were purchased from a grocery store. 

### 2.1. Meat Analog Preparation

Plant protein-based meat analog (MA) is preferred for different reasons including health, ethics, religion, animal welfare, natural resources, greenhouse gas emission, market price, etc. Plant protein-based meat analog was formulated with soy protein isolate (SPI), wheat gluten (WG), distilled water (DW) and canola oil (CO). At first, wheat gluten was mixed with fresh canola oil, and as soon as homogeneous slurry was obtained, soy protein isolate was added and mixed thoroughly. Then, water was added and mixed properly until a very soft-textured dough was developed. Respective proportions (g) of the ingredients WG: CO: SPI: DW were 12:15:22:73 (weight basis). Prepared dough was used to fill individual rectangular-shaped cavities of silicon mold (Freshware CB-115RD 24-Cavity Silicone Mini Mold). Covered silicon mold was held in a completely horizontal position (surrounded by water, but not immersed) on top of a flat metal wire surface and heated for 15 min at 70 °C in a water bath. After heat treatment, the mold was allowed to reach equilibrium with room temperature. After that, gentle pressure (using finger) was applied at the back of each cavity to bring out the formed substrate without any fracture. This substrate was used as meat analog (MA), with an average weight of 16 ± 0.2 g/substrate and a uniform dimension of 5.6 cm × 2.5 cm × 1.3 cm. Prepared MA was packed in zip lock bags and stored in a refrigerator (4 ± 2 °C) for 24 h, before its use. 

### 2.2. Batter Coating

The use of flour-based batter coatings on food substrates is a popular method of producing fried products with low fat. To prepare batter coatings: crystals of sodium pyrophosphate were ground to obtain a fine powder. Then, sodium bicarbonate (1.4%), NaCl (1.5%) and methylcellulose (0.3%) were added and mixed properly with sodium pyrophosphate powder (1.8%) to form a homogeneous powder mix. The powder mixture was thoroughly mixed with dry flour in a proportion of 95% (flour) to 5% (powder mix). Distilled water (at chilled condition) was added gently to the solid ingredients with a total solid to water ratio of 1:1.3 and mixed properly until a homogenous tempura batter slurry was obtained. Rice flour-based batter (RB) was formulated using only rice flour, whereas wheat flour-based batter (WB) was formulated with wheat flour. Equal proportions of flours (rice and white) were used to formulate rice and wheat flour-based batter (RWB). The prepared batter systems were kept at room temperature (25 °C) for 5 min before being used to coat the MA. The MA was brought out of the refrigerator and kept at room temperature for 30 min prior to batter coating. It was fully immersed in the batter system for 1 min and then removed from batter solution to make a complete coat. The coated product was held on a kitchen fork for about 30 s to drain the excess amount, in order to obtain a uniform layer of coating over the meat analog (MA). The noncoated (NC) meat analog was used as a control sample. 

### 2.3. Parfrying and Freezing 

A programmable deep fat fryer (T-fal Compact, FF122851, made in China) filled with 1.5 L of fresh canola oil was used to partially fry the meat analog samples. The oil was preheated for 1 h and stirred to minimize any variation in oil temperature. Samples were uninterruptedly fried for 1 min at 180 °C, maintaining a sample to oil ratio of 1:30. At the end of parfrying, the frying basket was removed from oil and shaken 5 times. Meat analog-based parfried samples were allowed to cool at room temperature. Parfried samples were packed in Ziplock bag and kept at −18 °C for 7 days, and frozen products were used for subsequent finish-cooking treatment.

### 2.4. Finish-Cooking

Microwave heating (MH), infrared heating (IH), air frying (AF) and deep-fat frying (DFF) were used as finish-cooking methods for meat analog (MA)-based parfried frozen products. The internal core temperature of samples was considered a critical parameter for configuring operational settings of the finish-cooking methods. Finish-cooking methods were aimed at achieving a temperature ≥ 75 °C at the coldest point (at geometric center) within the samples. After each finish-cooking treatment, samples were left to rest at room temperature for 1 min, before further studies.

#### 2.4.1. Microwave Heating (MH)

The MA-based parfried frozen samples were heated using a microwave facility (Hamilton Beach, EM720CPN, China) operating at 2450 MHz/700 W. Actual microwave output power was 640 W, as determined using an IMPI 2-L test [18]. Parfried frozen samples were placed on the center region of high absorbent paper-based (Royale^®^, Toronto, ON, Canada) sandwich-like system, where six individual layers of absorbent paper were closely attached to each side of the samples. The whole system was placed on a dry and horizontally rotating (360°) glass structure in a microwave treatment chamber. The MH system was programed to provide uninterrupted heating for a duration of 1.5 min wherein the sample was exposed to a microwave power density (PD) of 25 W·g^−1^. The microwave PD was computed based on the measured initial weight of the sample and the actual power output of the microwave heating system, as detailed by Ngadi et al. (2009) and Kang and Chen (2015) [12,18].

#### 2.4.2. Infrared Heating (IH)

A programmable infrared facility (Toast-R-Oven, TO1380SKT, made in China) was used to perform the infrared heating (IH) of meat analog-based parfried frozen samples. Frozen parfried samples were placed on a stainless steel sample holder (39 × 15.5 cm), and the holder was placed in between of two cylindrical IR lamps (1150 W). The distance between the sample and IR lamps was adjusted to 8.9 cm and the sample was exposed to a heat flux of 1.9 W·cm^−2^. To achieve and maintain constant IR heat flux, the IR heating unit was turned on 5 min before starting the finish-cooking treatment following the relevant study [14]. The IH system was programmed to maintain a constant temperature of 180 °C for a total duration of 15 min and the sample was flipped over at halfway point. 

#### 2.4.3. Air Frying (AF)

A programable air fryer unit (Philips HD9240/90, China) was used for air frying (AF) of the MA-based parfried frozen samples. To reach thermal equilibrium, AF unit was turned on for 5 min before its use to fry the parfried frozen samples. Frozen parfried samples were placed in the bucket (on top of wire mesh) of AF unit. The AF unit was programmed to fry the samples at a constant temperature of 180 °C for an uninterrupted duration of 15 min. There was no need to rotate/flip over the samples during AF, since hot air covered all parts of the samples’ surfaces. At the end of AF, samples were removed from frying basket and used for further studies. 

#### 2.4.4. Deep-Fat Frying (DFF)

Deep-fat frying (DFF) of meat analog-based parfried frozen samples was performed at atmospheric pressure (1 atm) in a programmable fryer (T-fal Compact, SERIE F53-S3, China) filled with 1.5 L fresh canola oil. To maintain the set temperature of frying oil, the DFF unit was turned on 1 h before starting the frying experiment. Parfried frozen samples were fried at 180 °C for 3 min, maintaining a sample to oil ratio of 1:30. It was cautiously monitored that the samples were fully immersed in oil during the full length of DFF. At the end of DFF, frying basket was removed from oil and shaken 5 times. Each batch of used frying oil was replaced with a new batch of fresh oil after thirty minutes of frying. The finish-fried samples were used for further studies without any prior treatment. 

### 2.5. Process Parameters 

Batter pickup (BP) was calculated to determine the amount of raw batter that adhered to meat analog. The BP was calculated as follows:

(1) $$\mathrm{batter}\;\mathrm{pickup},\;\mathrm{BP}\;(\%)=\frac{wt.\;of\;MA\;after\;batter\;coating-wt.\;of\;MA\;before\;batter\;coating}{wt.\;of\;MA\;before\;batter\;coating}\times 100$$

Parfrying loss (PL) determined total weight loss and was calculated by dividing the weight difference as follows:

(2) $$\mathrm{parfrying}\;\mathrm{loss},\;\mathrm{PL}\;(\%)=\frac{wt.\;of\;MA\;before\;parfrying-wt.\;of\;MA\;after\;parfrying}{wt.\;of\;MA\;before\;parfrying}\times 100$$

In order to determine finish-cooking loss (F_CL_), parfried frozen MA samples were weighed before finish-cooking (Wi) and after finish-cooking (Wf) following the method of Rahimi et al. [14]. F_CL_ was calculated as follows:

(3) $$F_{\mathrm{CL}}\;(\%)=\frac{Wi-Wf}{Wi}\times 100$$

### 2.6. Physiochemical Properties

Color properties were assessed using a spectrophotometer (Minolta Spectrophotometer CM-3500d, Tokyo, Japan). Color was determined with CIELab space (Illuminant D65, 10° viewing angle) from a reflection spectra between 400 and 700 nm. Color parameters L (lightness–darkness), a* (greenness–redness), and b* (blueness–yellowness) were estimated at room temperature (25 °C) and total color difference (ΔE) was calculated as follows:

total color difference, ΔE = [ΔL^2^ + Δa^2^ + Δb^2^]^1/2^ (4)

where, ΔL = L_pf_ − L_fc_, Δa = a_pf_ − a_fc_, Δb = b_pf_ − b_fc_, _pf_ is parfried, and _fc_ is finish-cooked.

Textural attributes of the MA-based samples’ crusts were evaluated with a puncture test, using a mechanical texture analyzer (Stable Micro Systems Texture Analyzer, TA. HD PlusC, Surrey, UK). Test samples were individually mounted on a flat rigid support. A puncture probe TA-52 (2 mm dia.) applying 500 N was used to punch test samples at a constant test speed of 1 mm/s and a travel distance of 5 mm. A puncture test was performed at three equidistant locations of each individual sample. Following the methodology of Rahimi et al. [19], the maximum force to break (MF, N), displacement at maximum force (MD, mm), and slope (S, N/mm) at maximum force of the puncture test data were chosen as parameters to evaluate textural properties namely, hardness, brittleness and crispness, respectively. Puncture test data were analyzed using Exponent ver. 6.1.14 software (TA Instrument, Surrey, UK).

Moisture content was grouped into two categories namely, crust moisture (CrM) and total moisture (TM). CrM was defined as the moisture that was present in the outer crust region and TM includes both the core and crust moisture. The crust region of a finished-cooked sample was carefully separated from the core region using a microtome blade (Feather C35, Tokyo, Japan). The detached portion was freeze dried in a freeze dryer (Modulyod-115; Thermo Savant, New York, NY, USA) at −50 °C and 250 mbar for 48 h. Weight of the sample before and after freeze drying was measured, and moisture content was calculated on dry basis (g/g dry matter) by dividing the mass of moisture by the mass of the freeze-dried sample. Separately, some samples were freeze dried without separating crust from core, to measure total moisture.

To measure crust thickness (Ct), a cork borer was used to obtain flat portions from the detached crust, and Ct was measured using a calibrated digital slide-caliper. 

Freeze-dried samples were ground to increase the surface to volume ratio and an amount of 3–5 g was placed in thimbles of a VELP SER 148 solvent extraction unit (Velp Scientifica, Usmate, Italy). Fat was extracted with petroleum ether following the protocol of AOAC 960.39 (AOAC 1990). Weight of the extracted fat was measured. Fat content was computed on dry basis (g/g dry matter) by dividing the mass of extracted oil by the mass of freeze-dried sample.

### 2.7. Differential Scanning Calorimetry 

Glass transition temperature (Tg) of the detached crust of finish-cooked samples were determined using a differential scanning calorimeter (DSC Q250, TA Instrument, New Castle, DE, USA). Tg of samples was determined following the study of Rahimi et al. [19]. In brief: around 10–15 mg crust sample was placed in sample pan, a lid was attached, and a mechanical device was used to hermitically sealed them. One sample holder with a lid (but without crust sample) was used as a reference pan. In DSC measurement chamber: sample was rapidly cooled to −40 °C at a cooling rate of 20 °C/min, held isothermally for 2 min, and then heated to +40 °C at a rate of 2 °C/min. DSC data were recorded and analyzed using DSC-Trios ver.5.1.1 software (TA Instrument, New Castle, DE, USA). From DSC thermogram, Tg was obtained as onset of the heat capacity change and was represented as Tg_onset_. The Tg_onset_ was obtained as the change of the baseline slope, after a variation of heat flux occurred, and determined as the intersection of tangents to the transition curve.

### 2.8. ATR-FTIR Spectroscopy

An attenuated total reflectance Fourier transform infrared (ATR-FTIR) spectrometer (Nicolet iS5 FTIR, Thermo Fisher Scientific, Madison, WI, USA) was used to record the FTIR spectra of the surface of parfried samples. The operation of the spectrophotometer, acquisition and manipulation of spectra were performed using Omnic software (Nicolet 6.1v., Madison, WI, USA). The outer surface of the chosen sample was in direct contact with an ATR crystal, and spectra acquisition was performed at ambient temperature (25 °C). The ATR crystal was cleaned with ethanol to remove any residue (especially fat) from the previous sample. FTIR spectra were collected over the wavenumber range of 4000–950 cm^−1^ by co-adding 32 scans and at a resolution of 4 cm^−1^. All spectra were normalized against the background of air spectrum. After every 1 h of operation, a new reference air background spectrum was taken. The spectra were recorded as absorbance values. According to Chen et al. [20] and our preliminary study, absorption peaks at around 2922 cm^−1^ and 2852 cm^−1^ (asymmetrical and symmetrical stretching of –CH_2_), 1743 cm^−1^ (C=O stretching), and 1157 cm^−1^ (-C-O stretch; -CH_2_ bending) of the FTIR spectra were considered a surface fat response, i.e., the presence of frying canola oil on a crust surface. From this view, considering pertinent studies [21,22,23], absorption area under the spectral ranges 1130–1200 cm^−1^, 1425–1475 cm^−1^, 1700–1775 cm^−1^, and 2800–3000 cm^−1^ were calculated using Omnic software (6.1v), and their summed value was reported as a surface fat response (SFR, au). According to Nicolaisen (2009) and Efimov et al. (2003), a broad peak around 3000–3700 cm^−1^ was due to the components of asymmetric and symmetric stretching modes of the H_2_O molecule [24,25]. An absorption area in FTIR spectra within this (3000–3700 cm^−1^) range as well as an absorption region between 1525 and 1725 cm^−1^ was considered a surface moisture response (SMR, au).

### 2.9. Scanning Electron Microscopy

Scanning electron microscopy (SEM) was used to assess surface morphology. Surface-washed (with three consecutive charges of petroleum ether, for a total immersion period of three min) samples were freeze dried and used for SEM imaging with a scanning electron microscope (Hitachi TM3000, Tokyo, Japan). SEM operational procedure of Adedeji and Ngadi [7] was followed with required modifications. In brief: 5 kV electron power, auto contrast function and composition mode of imaging were used. A small cut, rectangular-shaped, defatted sample of about 10 × 10 × 5 mm in dimension was placed on the sample base with a sticky surface that comprises carbon. After each sample was properly aligned using visual control with two sets of knobs and images shown in the control software on a computer screen, vacuum pressure was created in the sample chamber and images were quickly acquired to prevent charging and heating that could lead to artifacts in the images. The SEM images were acquired at a magnification of 30× and recorded as an 8-bit joint photographic experts group (JPEG) file. To characterize the surface roughness, the fractal dimension (FD) of the surface was estimated from 2D SEM images using the box-counting method, as detailed by Rahimi and Ngadi [26].

### 2.10. Statistical Analysis

All experimental data obtained from the triplicate of samples’ mean ± standard deviation were reported. Tukey’s HSD (honestly significant difference) was used for reporting significant (*p* < 0.05) differences among means. Statistical analyses were performed using a licensed statistical software, JMP 14.1v (SAS Institute Inc., Cary, NC, USA). 

## 3. Results and Discussion

**Figure 1:**
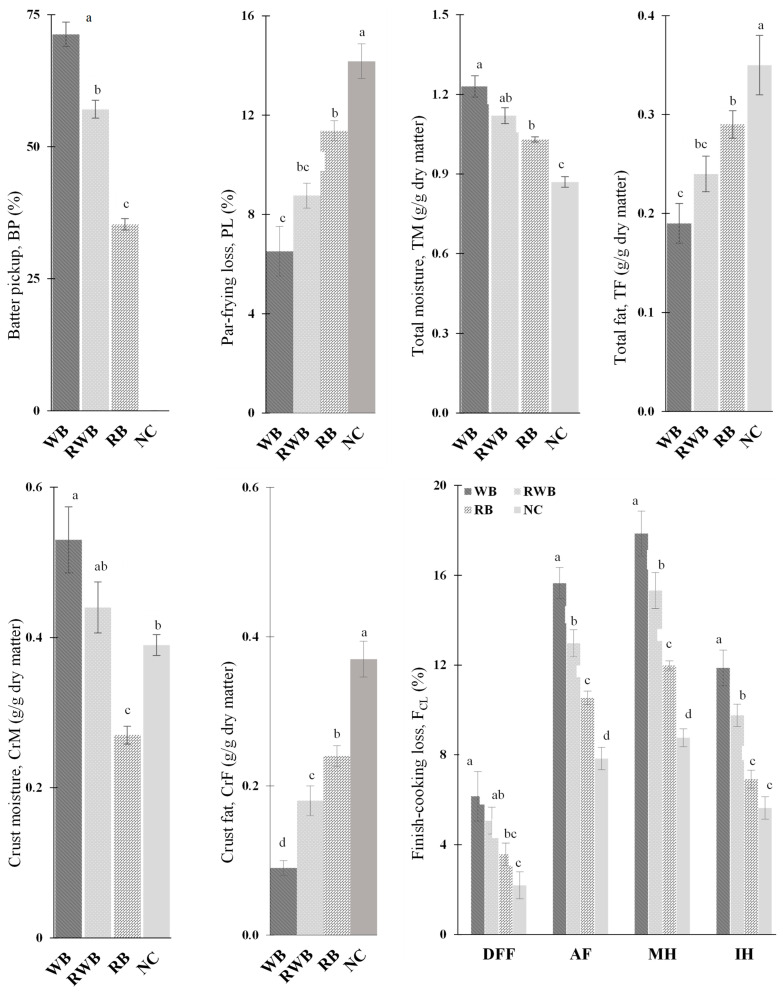
Process parameters and attributes of parfried sample. NC, RB, RWB and WB represent noncoated, rice flour-based-batter-coated, wheat and rice flour-based-batter-coated, and wheat flour-based-batter-coated meat analog, respectively. DFF, AF, MH and IH represent deep-fat frying, air frying, microwave heating and infrared heating, respectively. Lower-case letters (a–d) indicate significant (*p* < 0.05) differences in values for a given event.

Figure 1 depicts the process parameters and attributes of the parfried samples. The average batter pickup (BP) by plant protein-based meat analog (MA) was 71.3% for wheat-flour-based batter (WB) and 35.3% for rice flour-based batter (RB). Lower (6.51% < 11.37%) parfrying loss (PL) and concomitant higher (0.53 > 0.27 g/g dry mass) crust moisture (CrM) and (1.23 > 1.03 g/g dry mass) total moisture (TM) were determined for WB-batter-coated meat analog, whereas higher (0.24 > 0.09 g/g dry mass) crust fat (CrF) and higher (0.29 > 0.19 g/g dry mass) total fat (TF) were determined for RB-batter-coated meat analog. These differences could be explained as such: wheat flour contains a higher amount of protein (gluten) that can form 3D networks during frying which prevents moisture loss and fat uptake; the methylcellulose was in favor of the higher viscosity and moisture retention ability of wheat flour-based batter [9,27]. These properties resulted in lower moisture loss from WB-coated meat analog and lower fat absorption during parfrying. The PL, CrF and TF of batter-coated parfried meat analogs were significantly (*p* < 0.05) lower than those of the noncoated (NC) control sample.

The finish-cooking loss (F_CL_) of parfried frozen meat analog was greatly dependent on the cooking method: the lowest F_CL_ (ranging from 2.19 to 6.15%) was observed for deep-fat frying (DFF). The highest F_CL_ (ranging from 8.76 to 17.85%) was observed for microwave heating (MH) followed by air frying (AF). The F_CL_ ranged from 5.63 to 11.17% for infrared heating (IH). These disparities can be explained as such: DFF is a simultaneous heat and mass transfer process in which the meat analog samples lost their moisture and absorbed frying-oil, which minimized the overall weight differences that were reflected as a lower F_CL_. Under IH, MH and AF treatments, meat analog samples only lost their moisture without absorbing any oil; as a consequence, a higher F_CL_ was observed. The contribution of oil leaching out (as drip loss) in favor of the weight loss of parfried frozen samples could also be a probable cause of a higher F_CL_. This could be understood as such: at a frozen temperature (−18 °C) the frying oil (absorbed during parfrying) was in a solid state and was strongly attached to the solid matrix of parfried MA samples. Under heat treatment, with a rise in temperature of the frozen samples, the adhered oil melts and start to flow, as the viscosity of oil reduces with an increase in temperature [28]. The highest F_CL_ under MH might be due to its rapid heat generation; the generated heat moved inner moisture and fat components out which were subsequently removed with the absorbent paper arrangements. The F_CL_ under MH could be controlled by modifying the power density (PD), i.e., sample volume to microwave power. Compared to IH, higher finish-cooking loss under AF treatment might be due to the intense removal of moisture and oil with high-velocity hot air, along with the drip loss phenomenon. Overall, higher F_CL_ was observed for WB-batter-coated meat analog samples in comparison to RB-coated samples. This could be linked with the presence of higher moisture in WB-coated parfried samples, as during the finish-cooking treatment of the parfried-frozen samples, the probable source of weight reduction was the loss of flowable components (i.e., moisture, fat). For the studied samples and methods of cooking, the finish-cooking loss of meat analog-based batter-coated parfried frozen products was less than 20%. Under IR treatments, weight loss of up to 27.3% has been reported for parfried chicken nuggets [14]. 

**Figure 2:**
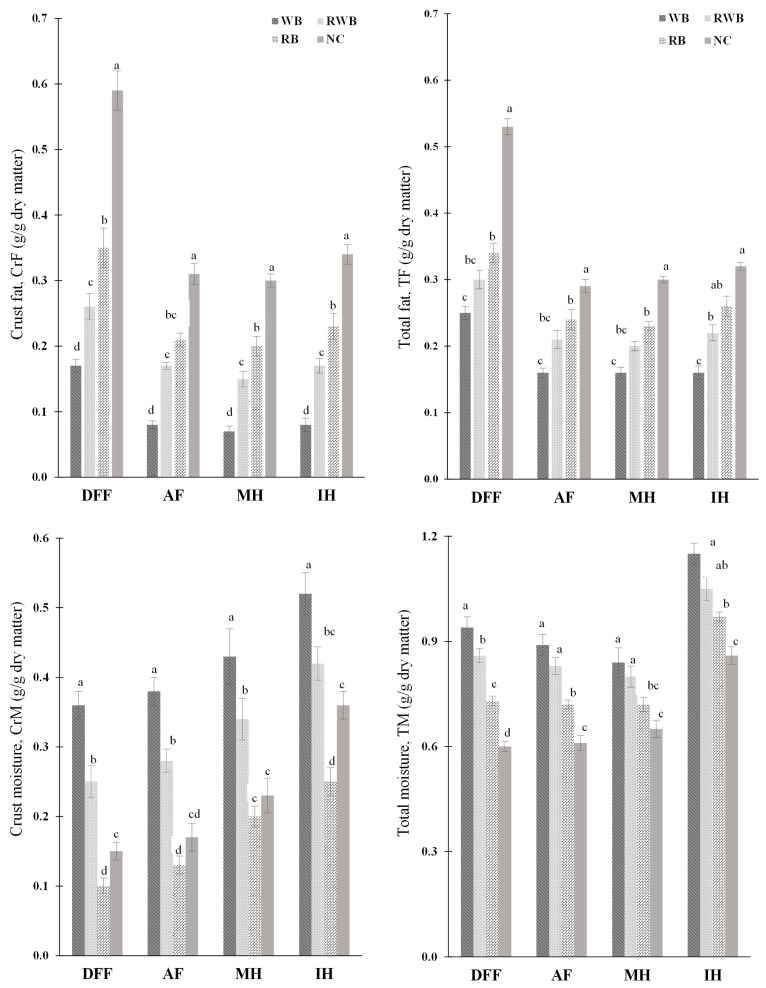
Moisture–fat profile of finish-cooked products. NC, RB, RWB and WB represent noncoated, rice flour-based-batter-coated, wheat and rice flour-based-batter-coated, and wheat flour-based-batter-coated meat analog, respectively. DFF, AF, MH and IH represent deep-fat frying, air frying, microwave heating and infrared heating, respectively. Lower-case letters (a–d) indicate significant (*p* < 0.05) differences in values for a given event.

Figure 2 represents a moisture–fat profile of finish-cooked products. Compared to DFF, both the crust fat (CrF) and total fat (TF) content of alternatively finish-cooked (via IH, MH, AF) meat analogs were substantially lower. Hence, MW, IH and AF could be considered as healthier alternatives to deep-fat frying (DFF) for cooking meat analog-based parfried frozen products. The fat content (g/g dry matter) of finish-cooked samples was either similar to or lower than the fat content of samples in their respective parfrying stage. In comparison to parfried samples, the presence of lower fat in finish-cooked products indicates oil leaching out as drip loss [14,15]. However, the higher fat profile for DFF was associated with a loss of moisture from parfried frozen samples and consequent oil uptake during finish-cooking in oil. In DFF, the use of a suitable oil/oil blend might be a way to reduce fat content in finish-cooked meat analog-based products, as the degree of saturation of frying oil affects the fat content of fried products [29]. In DFF, significantly (*p* < 0.05) lower fat was found in batter-coated samples in comparison to noncoated sample. However, fat content was significantly (*p* < 0.05) lower in WB-batter-coated products in comparison to RB-batter-coated products; this could be the effect of higher water retention and the fat-uptake prevention ability of wheat flour-based batter [9]. 

Moisture profiles of meat analog (MA)-based parfried frozen products were considerably impacted by the studied finish-cooking methods. The extent of moisture loss from parfried frozen products was interlaced with both finish-cooking methods and batter-formulations. Lower crust moisture (CrM) was observed for the DFF method of finish-cooking. Higher moisture loss in DFF is attributed to its higher rate of heat transfer, as frying is a simultaneous heat and mass transfer process in which mass profiles change with the application of heat [15]. Among other methods, a higher extent of total moisture (TM) loss was found for MH, followed by AF, and a lower extent of moisture loss was found for IH. A higher extent of moisture loss for MH was not surprising. The use of microwave power as a pre-treatment method to reduce initial moisture of chicken nuggets has been reported in the literature [12]. For identical meat analog-based samples, moisture in the crust region of food finish-cooked via DFF and AF were quite similar. However, for IH, the presence of high amount of moisture in the crust region was observed. Overall, the moisture content (CrM, TM) of noncoated meat analog-based products was significantly lower than that of their batter-coated counterparts.

**Figure 3:**
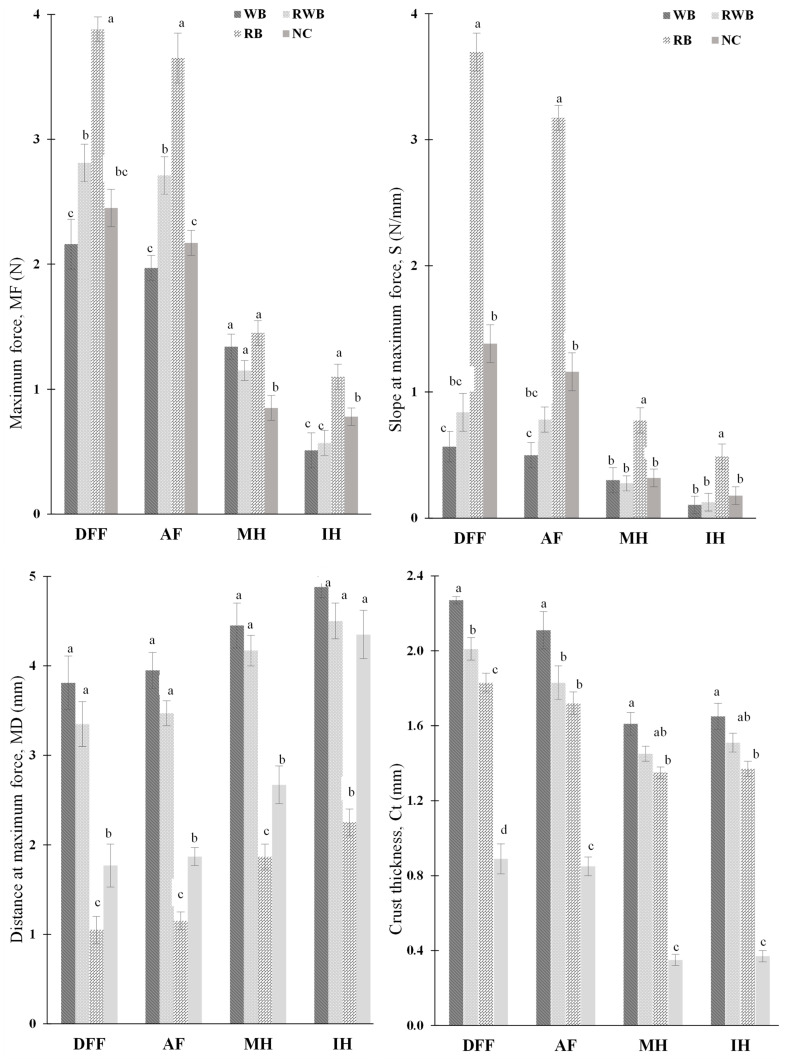
Textural and structural profile of finish-cooked samples. NC, RB, RWB and WB represent noncoated, rice flour-based-batter-coated, wheat and rice flour-based-batter-coated, and wheat flour-based-batter-coated meat analog, respectively. DFF, AF, MH and IH represent deep-fat frying, air frying, microwave heating and infrared heating, respectively. Lower-case letters (a–d) indicate significant (*p* < 0.05) differences in values for a given event.

Figure 3 shows the texture profile and crust thickness of finish-cooked products. Hardness (MF), ductileness (MD) and crispiness (S) of finish-cooked products were greatly impacted by the choice of finish-cooking method. Higher values of MF and S with a lower value of MD for the samples that underwent DFF and AF treatment indicate that both deep-fat frying and air frying produced crusts with a hard, brittle and crispy texture. This suggests that, to develop the desired textural attributes with lower fat content in meat analog (MA)-based finish-cooked products, air frying could be used as a suitable alternative finish-cooking method to conventional deep-fat frying. It is notable that the applied cooking time of AF was (15 min), considerably higher than the duration of DFF treatment (3 min). As AF-treated samples contained considerably lower fat, air frying could be used as a healthier alternative to deep-fat frying in preparation of MA-based batter-coated products. In the same context, both MH and IH could also be considered as healthier alternatives to deep-fat frying. In the case of IH and MH, modification of process parameters such as longer processing time (for IH), use of susceptor materials (for MH), etc., could be considered to achieve the desired textural attributes of finish-cooked products. However, textural changes that occur in foods during cooking are mainly due to the evaporation of water, protein denaturation and starch gelatinization [15]. In harmony with this phenomenon, a very similar moisture profile was observed when the samples were finish-cooked via DFF and AF (Figure 2). The presence of higher moisture in the crust region of IH- and MH-treated samples might be the prime cause of their less crispy texture, as moisture and crunchiness of foods are generally negatively correlated [19]. Compared to IH, MH produced a hard crust, but it was not brittle and crispy. As moisture removal was higher in MH, a hard crust developed as a result of the presence of lower moisture. However, crispiness development in battered products might not be solely dependent on their moisture content, as their spatial distribution and overall microstructure might also be influential contributors. Overall, textural attributes had a strong relationship with batter formulations: for identical cooking method, the hardness, brittleness and crispiness of RB-batter-coated products were significantly (*p* < 0.05) higher than WB-batter-coated samples. This disparity is associated with the flour composition of batter systems and final moisture content in the crust region (CrM). This can be understood as such: the starch content of parfried frozen batters gelatinized during heat treatments (during finish-cooking); when the samples were removed from the finish-cooking units, with the lowering of the temperature, the samples became hard, which might be due to the occurrence of starch retrogradation. In addition, starch (carbohydrates) content was higher in rice flour. Post-fry changes in the textural properties of starch-based batter-coated deep fat-fried potatoes has been reported in the literature [19]. 

The crust thickness (Ct) measurements of the DFF- and AF-treated products were similar, while those of IH- and MH-treated samples were in close similarity. This indicates that during finish-cooking treatments, the effective outer boundary layer (which produces crust) changed depending on the cooking method. This change was batter formulation-dependent; wheat flour-based batter produced a thicker crust, and rice flour-based batter produced a thinner crust. This property can explain the other physiochemical properties: a thicker WB crust facilitated higher moisture retention and prevented fat uptake, whereas due to presence of lower moisture, the RB crust was crispier. Therefore, modification of the formation of crust structure (via modification of batter formulation) could be a way to develop the desired textural properties in finish-cooked MA-based batter-coated products.

Table 1 summarizes post-finish-cooking moisture and texture attributes. At the post-finish-cooking stage, an increasing trend in crust moisture (CrM) was observed although total moisture (TM) was unchanged. The change in CrM was entangled with batter-formulation and finish-cooking method. Evolution in CrM indicates moisture redistribution between spatial region (from high moisture containing zone, to low moisture region), and increase in CrM is attributable to the moisture migration from inner core region of finish-cooked products. Post-finish-cooking moisture redistribution might be a continuous effect of finish-cooking, as moisture migration in a product can continue (due to vapor pressure difference) even after removal from the cooking unit [30]. A higher increase in CrM was observed in IH-treated samples. This could be linked with their higher initial total moisture content (TM), as moisture redistribution in food is generally considered as a diffusion-controlled phenomenon. Batter formulation influenced post-finish-cooking moisture redistribution behavior; a higher extent of moisture redistribution was observed in WB-batter-coated products in comparison to RB-batter-coated products. This could be attributed to the moisture absorption ability of fiber, as a good amount of fiber was present in wheat flour compared to rice flour (Section 2). 

Following the method of Rahimi et al. [19], post-finish-cooking changes in textural attributes were represented as normalized value of maximum force (MF*), normalized value of maximum distance (MD*) and normalized value of slope (S*). Textural attributes changed during the post-finish-cooking stage, wherein the nature of textural evolution varied between batter-formulations and finish-cooking methods. The DFF-processed samples were less prone to textural changes. This could be understood as such: major physicochemical transformations of parfried frozen, battered ingredients occurred under intense heat treatment (during finish-cooking with hot oil); consequently, their post-finish-cooking changes were lesser. AF-processed samples showed very similar behavior to that of the DFF-processed samples, as the processing time for AF was 15 min, thus its longer processing time might have compensated for the low heat intensity of air. However, the crust developed for RB batter was less prone to post-finish-cooking textural (crispiness, brittleness) changes, as this sample was mostly impacted during finish-cooking (shown by its higher initial values in Figure 3). The WB-batter-coated products’ crust showed proneness to post-finish-cooking textural changes that could be due to the higher extent of moisture redistribution between spatial regions. Interestingly, it was observed in RB-coated meat analog-based products that their hardness (MF) was prone to increase during the post-finish-cooking stage, especially when they had been finish-cooked via MH and IH. In contrast, the extent of post-finish-cooking textural change was lower in DFF- and AF-treated samples. This could be understood as such: during MH and IH, the starch in the batter coatings was gelatinized to a lower extent due to the shorter heating period and lower extent of heating, respectively; hence, remains undergo a higher extent of starch retrogradation during the post-finish-cooking stage and consequent changes in textural attributes were observed. Both DFF and AF might have intensely impacted the starch content of the batter systems during their finish-cooking stage; hence, post-finish-cooking textural changes were comparatively less. 

Table 2 summarizes glass transition temperature (Tg_onset_, °C) values of the meat analog-based finish-cooked products’ crusts. Tg values of the crusts were found to be in a negative temperature zone, ranging from −20 °C to −24 °C. In the literature [31], negative Tg has been reported for fried food matrixes such as carrot chips (−39 °C), donuts (−18 °C) and french fries (−11.8 °C). Tg values in a range between −15 and −21 °C have been reported for modified starch (potato, corn, tapioca)-based fried batters [19]. However, Tg values of finish-cooked products’ crusts were far lower than the post-finish-cooking holding temperature (25 °C) in this study. The difference between Tg and holding temperature explains the post-finish-cooking evolution of crust moisture and texture profile of the studied samples. Higher stability of the quality attributes is anticipated at a temperature below the intrinsic Tg of a matrix [32]. Mochizuki et al. [33] reported a very strong correlation between Tg and the hardness of a compressed soup solid. However, comparatively higher Tg might have supported the post-finish-cooking textural stability of DFF- and AF-processed samples, compared to other methods of finish-cooking. It is notable that a small difference in Tg could cause a large difference in viscosity (due to an increase in molecular mobility) and consequently affect the physical properties of the studied matrix [34,35]. 

**Figure 4:**
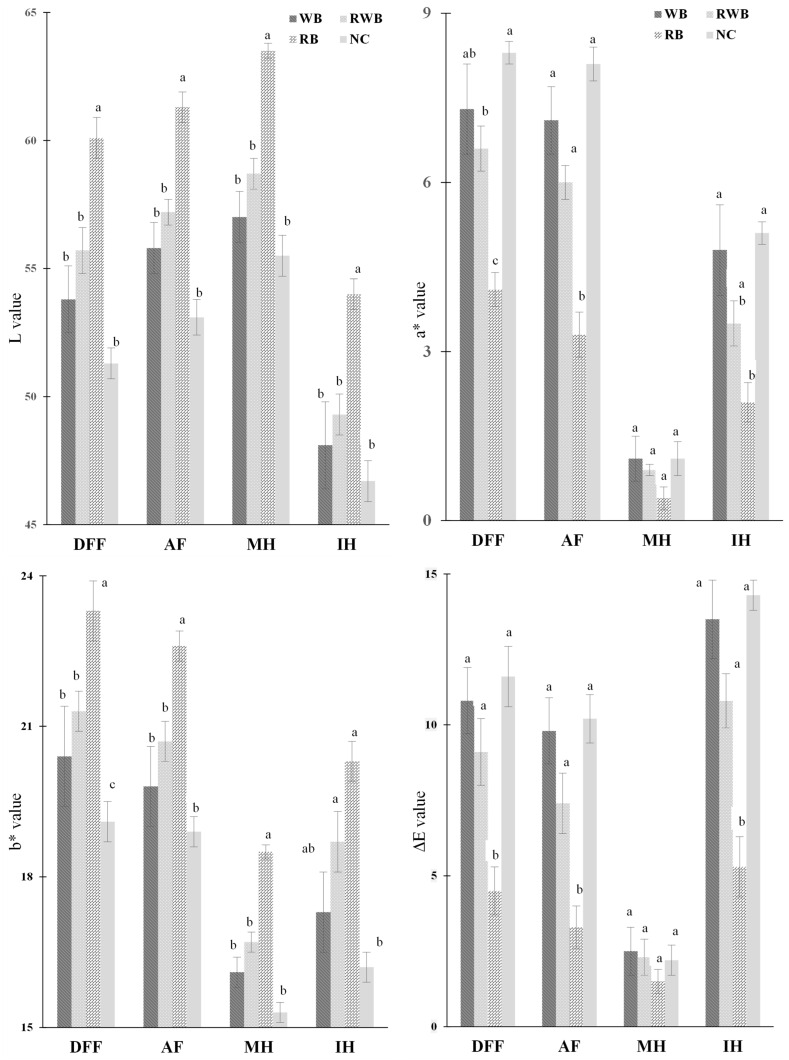
Color indices of finish-cooked samples. NC, RB, RWB and WB represent noncoated, rice flour-based-batter-coated, wheat and rice flour-based-batter-coated and wheat flour-based-batter-coated meat analog, respectively. DFF, AF, MH and IH represent deep-fat frying, air frying, microwave heating and infrared heating, respectively. Lower-case letters (a–c) indicate significant (*p* < 0.05) differences in values for a given event.

Color is very crucial for any food item as this is the quality parameter that is evaluated by consumers even before food enters the mouth. Figure 4 portrays the color attributes of finish-cooked products. Color traits (L, a*, b*) of AF-processed samples were quite similar to those of DFF-processed samples. This similarity in color attributes might be associated to their heat intensity and treatment duration. As such, rapid color evolution in DFF treatment was due to its high rate of temperature rise, and comparable color development in AF treatment was due to its longer treatment time. MH retained the initial color characteristics of parfried samples (represented by lower ΔE value), which were characterized by higher lightness (L), lower redness (a*) and lower yellowness (b*) color. The color of MH-processed finish-cooked products indicates that microwave heating caused less alteration to the surface color of parfried samples. To minimize the changes in color properties during finish-cooking, MH would be preferred over conventional DFF. However, IH caused the formation of an undesirable darker surface (lower L value). This could be due to its longer processing time and the direct exposure of IR rays on the food’s surface. Generally, food components absorbed electromagnetic energy and the color of the food became darker with a higher intensity IR treatment [14]. Overall, color attributes of meat analog-based finish-cooked products were greatly impacted by the formulations of batter coatings. L (lightness) and b* (yellowness) values of RB-batter-coated products were higher than those of WB-batter-coated products, whereas higher redness (a* value) was noticed for WB-coated products.

**Figure 5:**
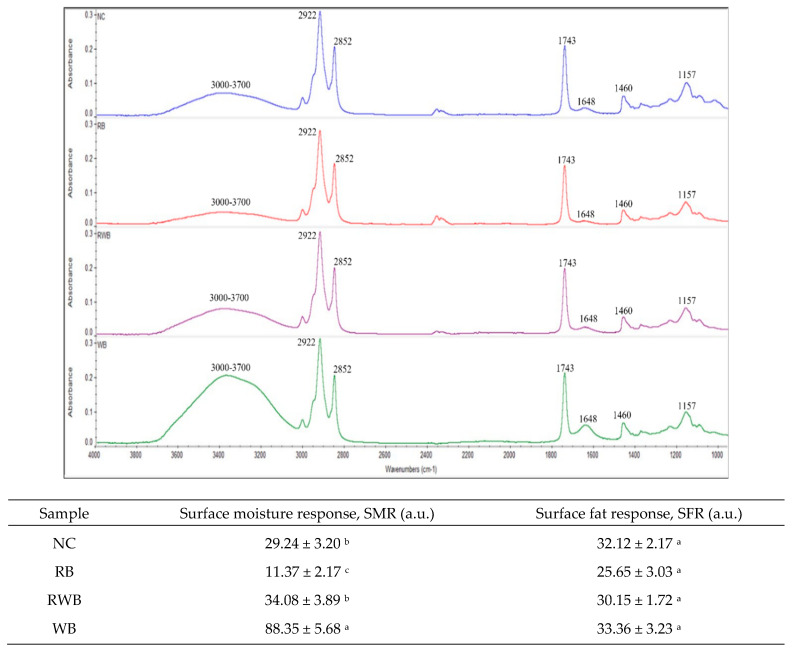
ATR-FTIR spectra of parfried samples and estimated mean values of absorbance unit. Downwards spectra: NC, RB, RWB and WB represent noncoated, rice flour-based-batter-coated, wheat and rice flour-based-batter-coated and wheat flour-based-batter-coated meat analog, respectively. Lower case letters (a–c) represent significant (*p* < 0.05) differences among means (within column).

Non-zero and higher ΔE values showed that the initial color of parfried frozen samples had undergone prominent changes due to IH, followed by DFF and AF. Compared to RB, a higher extent of total color change was observed in WB-coated samples. Parfried samples’ surface chemistry assessment using ATR-FTIR spectroscopy (Figure 5) provides more insight to understand the variation in color changes from finish-cooking processes. Absorption peaks around 2922, 2852, 1743, 1460, and 1157 cm^−1^ represent the canola oil on surface of parfried samples. The presence of broad peaks around 3000–3700 cm^−1^ and 1648 cm^−1^ in the spectra of WB indicates the presence of more surface moisture. The presence of more moisture might be a cause of its lower L value, as color development during frying occurs because of moisture loss. In addition, the presence of higher protein content in WB batter might facilitate the formation of a brown color via the Maillard reaction [14], as this reaction requires amino acids which were abundant in wheat flour (Section 2).

**Figure 6:**
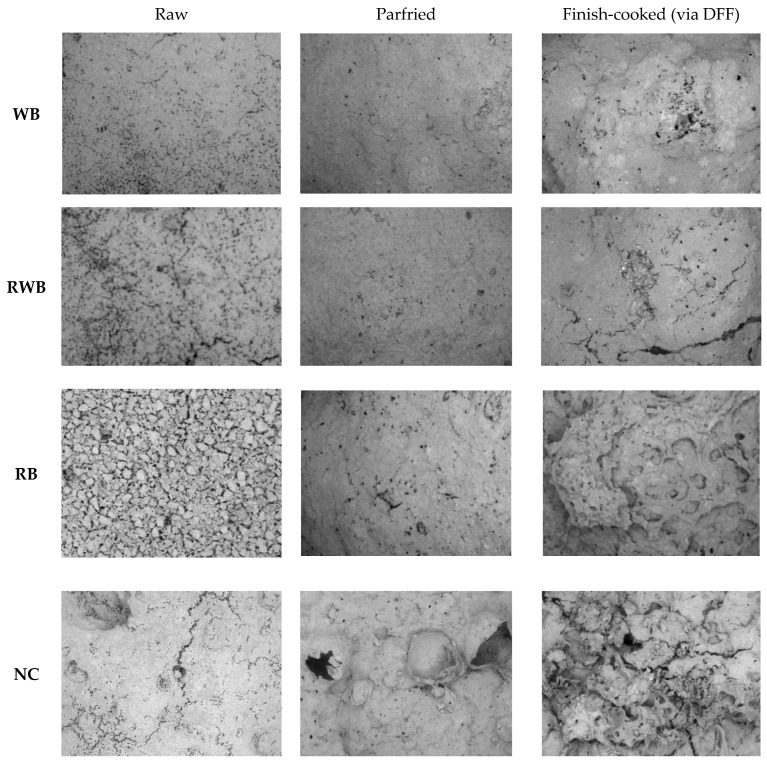
Processed micrograph of the meat analog-based samples’ surfaces. NC, RB, RWB and WB represent noncoated, rice flour-based-batter-coated, wheat and rice flour-based-batter-coated and wheat flour-based-batter-coated meat analog, respectively. DFF: deep-fat frying.

Figure 6 illustrates the evolution of the surface microstructures of the meat analog-based samples. Polygonal shape structures were very prominent in the scanning electron microscopy (SEM) image of the raw RB-coated sample; these polygonal structures might be the starch granules of raw rice flour [7]. In the SEM image of the parfried RB sample these polygonal shapes were not present, and a comparatively flat surface was observed; this change could be understood as the gelatinization of starch. However, due to parfrying, the surface of WB and RWB batter had become smooth. This might be due to the denaturation of their protein entities under heat treatment. Overall, the parfrying process created surface irregularities such as holes, ruptures and crevices. The presence of these structural irregularities was mostly noticed in parfried RB batter. These surface structures have acted as access ways for moisture loss and consequent fat uptake during finish-cooking via DFF. A loss of moisture and a loss of oil were observed under AF, MH and IH treatments. A surface micrograph of the finish-cooked, noncoated (NC) meat analog sample showed a higher extent of these structural irregularities. This explains their higher moisture loss and consequent higher fat uptake during deep-fat frying treatment (discussed earlier).

**Figure 7:**
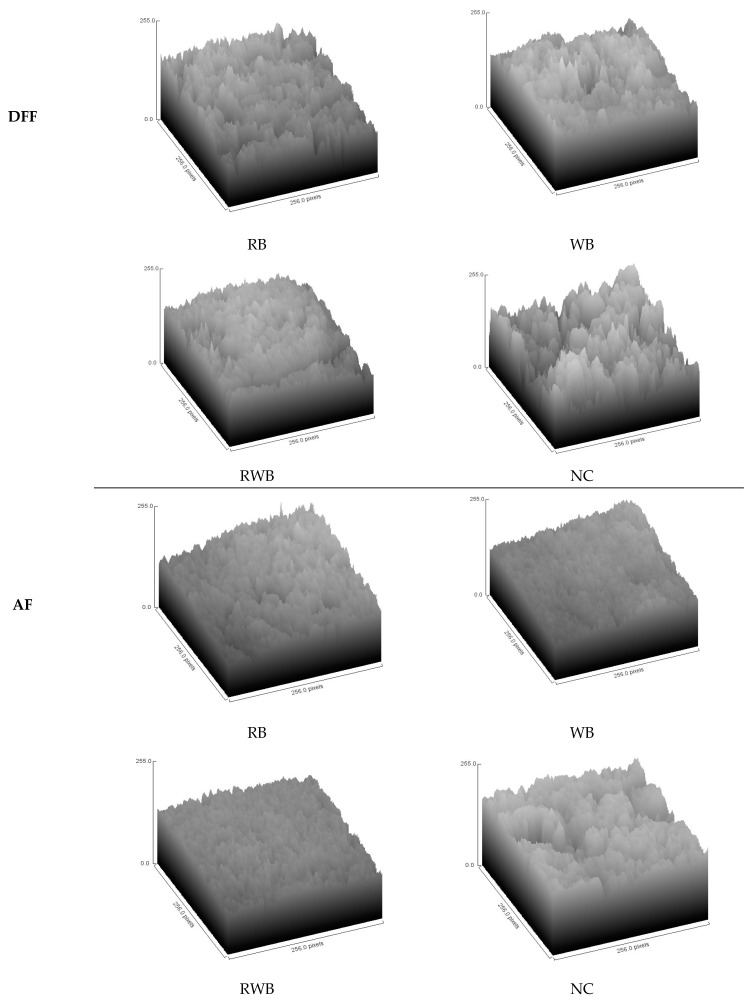
Surface plot depicting roughness of finish-cooked meat analog-based products. NC, RB, RWB and WB represent noncoated, rice flour-based-batter-coated, wheat and rice flour-based-batter-coated and wheat flour-based-batter-coated meat analog, respectively. DFF: deep-fat frying. AF: air frying.

Surface roughness of the food matrix is a crucial parameter relating to its consumer acceptance. Figure 7 represents the surface roughness of meat analog-based finish-cooked products. Table 3 summarizes the effects of batter formulations and finish-cooking methods on surface roughness, as evaluated using the estimated fractal dimension (FD) value. Higher surface roughness was observed for RB-batter-coated meat analog samples in comparison to WB-batter-coated samples, and the relatively higher surface roughness reflected a higher fractal dimension (FD) value of RB coated samples. Batter formulation-associated differences in fried matrixes’ surface microstructure have been reported in the relevant literature [26]. Overall, the surface roughness of parfried samples increased (indicated by higher FD) due to finish-cooking, and deep-fat frying (DFF) caused higher surface roughness in comparison to air frying (AF).

## 4. Conclusions

Batter formulations greatly influenced the process parameters and attributes of meat analog (MA)-based coated food products. In comparison to deep-fat frying (DFF), microwave heating (MH), infrared heating (IH) and air frying (AF) resulted in a higher finish-cooking loss (F_CL_) of meat analog-based products. MH, IH and AF significantly reduced the spatial (crust, total) fat content of MA-based finish-cooked products, in comparison to DFF. Spatial (crust, total) moisture profiles of MA-based finish-cooked products were dependent on both batter formulations and cooking methods. The evolution of textural (hardness, brittleness, crispiness) and color attributes (lightness, redness, yellowness) of the MA-based coated products was associated with batter formulations and cooking methods. In terms of the fat content of finish-cooked meat analog-based products, the electromagnetic heating (MH, IH) processes could be considered healthier alternatives to DFF. In terms of textural attributes, the latter method was preferable over the former two. Overall, the AF technique has shown great potential to be used as a very suitable substitute for DFF to finish-cook meat analog-based, batter-coated, parfried frozen food products. It could be suggested that the use of the AF technique in industrial/food service points would offer a dual benefit (i.e., health and economic), as this technique requires no oil for preparing food.

## Figures and Tables

**Table 1:** Post cooking evolution of moisture and texture profile.

Cooking Method	Sample	Moisture Content (g/g Dry Matter)				Textural Attribute (Normalized)					
		CrM		TM		MF *		MD *		S *	
		0 min	30 min	0 min	30 min	0 min	30 min	0 min	30 min	0 min	30 min
DFF	WB	0.36 ± 0.03 ^a^	0.40 ± 0.02 ^a^	0.94 ± 0.03 ^a^	0.92 ± 0.02 ^a^	1.0 ^A^	0.87 ± 0.03 ^B^	1.0 ^A^	0.90 ± 0.02 ^B^	1.0 ^A^	0.92 ± 0.03 ^B^
	RWB	0.25 ± 0.02 ^b^	0.28 ± 0.03 ^b^	0.86 ± 0.02 ^b^	0.85 ± 0.03 ^b^	1.0 ^A^	0.90 ± 0.02 ^B^	1.0 ^A^	0.92 ± 0.03 ^B^	1.0 ^A^	0.94 ± 0.01 ^B^
	RB	0.10 ± 0.01 ^d^	0.11 ± 0.02 ^d^	0.73 ± 0.01 ^c^	0.73 ± 0.02 ^c^	1.0 ^B^	1.07 ± 0.02 ^A^	1.0 ^A^	1.02 ± 0.02 ^A^	1.0 ^A^	1.01 ± 0.02 ^A^
	NC	0.15 ± 0.02 ^c^	0.17 ± 0.02 ^c^	0.61 ± 0.01 ^d^	0.61 ± 0.02 ^d^	1.0 ^A^	0.98 ± 0.02 ^A^	1.0 ^A^	0.98 ± 0.02 ^A^	1.0 ^A^	0.97 ± 0.02 ^A^
AF	WB	0.38 ± 0.03 ^a^	0.43 ± 0.03 ^a^	0.89 ± 0.02 ^a^	0.88 ± 0.04 ^a^	1.0 ^A^	0.85 ± 0.03 ^B^	1.0 ^A^	0.89 ± 0.02 ^B^	1.0 ^A^	0.91 ± 0.03 ^B^
	RWB	0.28 ± 0.02 ^b^	0.32 ± 0.02 ^b^	0.83 ± 0.02 ^b^	0.81 ± 0.02 ^b^	1.0 ^A^	0.89 ± 0.02 ^B^	1.0 ^A^	0.91 ± 0.03 ^B^	1.0 ^A^	0.93 ± 0.02 ^B^
	RB	0.13 ± 0.01 ^d^	0.14 ± 0.03 ^d^	0.72 ± 0.01 ^c^	0.70 ± 0.03 ^c^	1.0 ^B^	1.09 ± 0.03 ^A^	1.0 ^A^	0.98 ± 0.02 ^A^	1.0 ^A^	0.98 ± 0.02 ^A^
	NC	0.17 ± 0.03 ^c^	0.29 ± 0.02 ^c^	0.61 ± 0.02 ^d^	0.60 ± 0.02 ^d^	1.0 ^A^	0.96 ± 0.03 ^A^	1.0 ^A^	0.97 ± 0.03 ^A^	1.0 ^A^	0.96 ± 0.03 ^A^
MH	WB	0.43 ± 0.05 ^a^	0.46 ± 0.04 ^a^	0.84 ± 0.03 ^a^	0.82 ± 0.04 ^a^	1.0 ^A^	0.89 ± 0.03 ^B^	1.0 ^A^	0.93 ± 0.03 ^B^	1.0 ^A^	0.89 ± 0.04 ^B^
	RWB	0.34 ± 0.04 ^a^	0.37 ± 0.03 ^a^	0.80 ± 0.02 ^ab^	0.79 ± 0.03 ^ab^	1.0 ^A^	0.93 ± 0.02 ^B^	1.0 ^A^	0.96 ± 0.04 ^B^	1.0 ^A^	0.91 ± 0.02 ^B^
	RB	0.20 ± 0.02 ^b^	0.21 ± 0.02 ^b^	0.72 ± 0.03 ^b^	0.71 ± 0.03 ^b^	1.0 ^B^	1.21 ± 0.05 ^A^	1.0 ^B^	1.05 ± 0.01 ^A^	1.0 ^A^	0.96 ± 0.04 ^A^
	NC	0.23 ± 0.04 ^b^	0.27 ± 0.03 ^b^	0.65 ± 0.02 ^c^	0.62 ± 0.03 ^c^	1.0 ^A^	0.95 ± 0.02 ^B^	1.0 ^A^	0.91 ± 0.06 ^B^	1.0 ^A^	0.92 ± 0.02 ^B^
IH	WB	0.52 ± 0.03 ^a^	0.59 ± 0.02 ^a^	1.15 ± 0.02 ^a^	1.15 ± 0.04 ^a^	1.0 ^A^	0.80 ± 0.02 ^B^	1.0 ^A^	0.85 ± 0.04 ^B^	1.0 ^A^	0.85 ± 0.03 ^B^
	RWB	0.42 ± 0.02 ^b^	0.48 ± 0.03 ^b^	1.05 ± 0.02 ^b^	1.04 ± 0.03 ^b^	1.0 ^A^	0.84 ± 0.03 ^B^	1.0 ^A^	0.89 ± 0.03 ^B^	1.0 ^A^	0.89 ± 0.02 ^B^
	RB	0.25 ± 0.01 ^d^	0.29 ± 0.02 ^d^	0.97 ± 0.01 ^c^	0.95 ± 0.03 ^c^	1.0 ^B^	1.13 ± 0.03 ^A^	1.0 ^A^	0.97 ± 0.03 ^A^	1.0 ^A^	0.96 ± 0.04 ^A^
	NC	0.36 ± 0.02 ^c^	0.38 ± 0.03 ^c^	0.86 ± 0.03 ^d^	0.84 ± 0.04 ^d^	1.0 ^A^	0.90 ± 0.02 ^B^	1.0 ^A^	0.93 ± 0.02 ^B^	1.0 ^A^	0.91 ± 0.03 ^B^

Lower-case letters (a–d) rank significant differences in moisture content among samples under same finish-cooking method and time. Upper-case letters (A–B) rank significant differences in the same textural attribute of the samples at different holding times. NC, RB, RWB and WB represent noncoated, rice flour-based-batter-coated, wheat and rice flour-based-batter-coated, and wheat flour-based-batter-coated meat analog, respectively. DFF, AF, MH and IH represent deep-fat frying, air frying, microwave heating and infrared heating, respectively. * represents normalized value.

**Table 2:** Glass transition temperature (Tg_onset_, °C) of finish-cooked meat analog.

Cooking Method	WB	RWB	RB	NC
DFF	−22.04 ± 0.19 ^c^	−21.14 ± 0.28 ^bc^	−21.01 ± 0.24 ^b^	−20.09 ± 0.22 ^a^
AF	−22.23 ± 0.28 ^c^	−21.46 ± 0.29 ^b^	−21.14 ± 0.23 ^b^	−20.18 ± 0.25 ^a^
MH	−22.86 ± 0.24 ^c^	−21.62 ± 0.33 ^b^	−21.18 ± 0.28 ^ab^	−20.32 ± 0.24 ^a^
IH	−23.11 ± 0.32 ^c^	−21.81 ± 0.32 ^b^	−21.34 ± 0.31 ^ab^	−20.51 ± 0.33 ^a^

NC, RB, RWB and WB represent noncoated, rice flour-based-batter-coated, wheat and rice flour-based-batter-coated and wheat flour-based-batter-coated meat analog, respectively. DFF, AF, MH and IH represent deep-fat frying, air frying, microwave heating and infrared heating, respectively. Lower-case letters (a–c) indicate significant (*p* < 0.05) differences in values for a given event (within row).

**Table 3:** Fractal dimension (FD) of meat analog-based products.

Sample	WB	RWB	RB	NC
Parfried	2.559 ± 0.016 ^b^	2.568 ± 0.014 ^b^	2.598 ± 0.012 ^a^	2.530 ± 0.031 ^c^
AF	2.588 ± 0.012 ^c^	2.597 ± 0.015 ^bc^	2.613 ± 0.011 ^ab^	2.644 ± 0.024 ^a^
MH	2.598 ± 0.027 ^c^	2.612 ± 0.018 ^c^	2.671 ± 0.006 ^b^	2.685 ± 0.014 ^a^
IH	2.613 ± 0.032 ^b^	2.658 ± 0.037 ^b^	2.704 ± 0.022 ^a^	2.738 ± 0.036 ^a^
DFF	2.827 ± 0.018 ^c^	2.854 ± 0.02 ^c^	2.921 ± 0.010 ^b^	2.948 ± 0.024 ^a^

NC, RB, RWB and WB represent noncoated, rice flour-based-batter-coated, wheat and rice flour-based-batter-coated and wheat flour-based-batter-coated meat analog, respectively. DFF, AF, MH and IH represent deep-fat frying, air frying, microwave heating and infrared heating, respectively. Lower-case letters (a–c) indicate significant (*p* < 0.05) differences in values for a given event (within row).

## Data Availability

The data that support the findings of this study are available from the corresponding author upon reasonable request.
